# Blood type, ABO genetic variants, and ovarian cancer survival

**DOI:** 10.1371/journal.pone.0175119

**Published:** 2017-04-27

**Authors:** Gabriella D. Cozzi, Rebecca T. Levinson, Hilary Toole, Malcolm-Robert Snyder, Angie Deng, Marta A. Crispens, Dineo Khabele, Alicia Beeghly-Fadiel

**Affiliations:** 1 Division of Epidemiology, Department of Medicine, Vanderbilt University Medical Center, Nashville TN, United States of America; 2 Vanderbilt Genetics Institute, Vanderbilt University School of Medicine, Nashville TN, United States of America; 3 Meharry Medical College, Nashville TN, United States of America; 4 Department of Obstetrics and Gynecology, Vanderbilt University Medical Center, Nashville TN, United States of America; 5 Vanderbilt-Ingram Cancer Center, Nashville TN, United States of America; National Cancer Institute, UNITED STATES

## Abstract

**Objective:**

Blood type A and the A1 allele have been associated with increased ovarian cancer risk. With only two small studies published to date, evidence for an association between ABO blood type and ovarian cancer survival is limited.

**Methods:**

We conducted a retrospective cohort study of Tumor Registry confirmed ovarian cancer cases from the Vanderbilt University Medical Center with blood type from linked laboratory reports and ABO variants from linked Illumina Exome BeadChip data. Associations with overall survival (OS) were quantified by hazard ratios (HR) and confidence intervals (CI) from proportional hazards regression models; covariates included age, race, stage, grade, histologic subtype, and year of diagnosis.

**Results:**

ABO phenotype (N = 694) and/or genotype (N = 154) data were available for 713 predominantly Caucasian (89.3%) cases. In multivariable models, blood type A had significantly better OS compared to either O (HR: 0.75, 95% CI: 0.60–0.93) or all non-A (HR: 0.77, 95% CI: 0.63–0.94) cases. Similarly, missense rs1053878 minor allele carriers (A2) had better OS (HR: 0.50, 95% CI: 0.25–0.99). Among Caucasians, this phenotype association was strengthened, but the genotype association was attenuated; instead, four variants sharing moderate linkage disequilibrium with the O variant were associated with better OS (HR: 0.62, 95% CI: 0.39–0.99) in unadjusted models.

**Conclusions:**

Blood type A was significantly associated with longer ovarian cancer survival in the largest such study to date. This finding was supported by genetic analysis, which implicated the A2 allele, although O related variants also had suggestive associations. Further research on ABO and ovarian cancer survival is warranted.

## Introduction

Ovarian cancer is the 5th leading cause of cancer deaths among women in the United States (US), with an estimated 22,280 new cases and 14,240 deaths in 2016 [1]. Despite improvements in chemotherapy and surgical cytoreduction (debulking) over the last twenty years, overall 5-year survival remains abysmally low at 45% [1]. Identifying additional prognostic factors and determining their contribution to disease outcomes could lead to new treatment approaches for women diagnosed with this typically fatal disease.

Landsteiner was awarded the Nobel Prize for his landmark discovery of blood types based on red blood cell (RBC) agglutination. In addition to transfusion medicine, blood type also gained a prominent role in genetics as one of the first traits with a Mendelian mode of inheritance and population-specific phenotypic variation. We now know that blood type is determined by genetic variation in the ABO gene on chromosome 9q34. The encoded glycosyltransferases catalyze the transfer of specific terminal oligosaccharides to the precursor H protein to form ABO antigens. Common variants result in different glycosyltransferases, oligosaccharide antigens, and phenotypes: N-acetylgalactosamine for blood type A, D-galactose for blood type B, both for blood type AB, and neither for an unmodified H antigen in blood type O. Blood type A and B differ predominantly by four amino acid substitutions (R176G, G235S, L266M, and G268A) from four common missense variants (rs7853989, rs8176743, r8176746, and rs8176747), while blood type O is predominantly due to a single nucleotide deletion (rs8176719) which shifts the reading frame and results in early protein termination [2–6]. In addition, multiple alleles, new mutations, and frequent recombination events add complexity to the genetic diversity of the ABO locus [3,4].

Blood type has been linked to multiple diseases, and genome-wide association studies (GWAS) have found associations between ABO variants and susceptibility to coronary artery disease (CAD), venous thromboembolism (VTE), and pancreatic cancer [7]. With regard to ovarian cancer, a meta-analysis conducted by the Ovarian Cancer Association Consortium (OCAC) demonstrated a small but significantly increased risk among women with genotypes conferring blood type A [8]. To our knowledge, only two small studies have evaluated ABO blood type and ovarian cancer survival. An early study published in Italian included 92 ovarian cancer cases and found 5-year survival rates of 64.5% and 33.3% for type O and A cases, respectively, although no multivariable analyses were conducted [9]. A more recent study of 256 Chinese women found that blood type A cases had two-fold shorter survival than non-A blood type cases in analyses adjusted for age, stage, and disease grade [10]. Multiple mechanisms have been postulated to explain the role of blood type in cancer progression, including altered cellular adhesion, immune response, and inflammation [11–13]. As current evidence is limited, we undertook this study to evaluate blood type, ABO genetic variation, and overall survival (OS) among women with ovarian cancer.

## Materials & methods

### Study population

After garnering appropriate Institutional Review Board approval (VUMC IRB #121299), Tumor Registry confirmed ovarian (C569) or fallopian tube (C570) cancer cases were identified using International Classification of Disease-Oncology (ICD-O) codes from de-identified electronic medical records (EMR) from the Vanderbilt University Medical Center (VUMC). EMR-linked Tumor Registry data included primary tumor site, histology, date of diagnosis, stage at diagnosis, and grade of disease. VUMC EMR data included date of birth and administratively-assigned race, which was comparable to self-reported race when evaluated in relation to genetic ancestry in a VUMC EMR study population [14]. Subject vital status was determined from both EMR and linkage to the National Death Index (NDI). Cases were considered to have died if they were listed as deceased in EMR or if there was a date of death from the NDI. Otherwise, OS was censored at the date of last EMR entry. A total of 208 of 1,328 unique subjects identified were excluded, leaving 1,120 Tumor Registry confirmed cases diagnosed between 1980 and 2013 for analysis. Exclusions included codes for a prior cancer diagnoses (N = 15), diagnosis before 1980 (N = 12) or after 2013 (N = 6), unknown diagnosis date (N = 16), or histology codes that indicated a germ cell tumor (9060, 9064, 9071, 9080, 9082, 9084, 9085), sex-cord stromal tumor (8620, 8634, 8640, 8670), or other non-epithelial tumor (8240, 8243, 8800, 8802, 8890, 8910, 9500, 9680). Cases with unknown histologic subtype were retained, as most were likely to be epithelial, but we did exclude cases with an age at diagnosis of less than 18 years (N = 3). In addition, cases with diagnosis and death occurring on the same day (N = 2), implausible survival times (N = 3), or lacking follow-up information (N = 58) were excluded.

### Blood type and ABO genotype

Blood type was ascertained from EMR-linked laboratory reports from serologic assays conducted on whole blood samples by the Vanderbilt Pathology Lab Service. EMR linked genetic data from the Illumina Exome BeadChip were also evaluated. Variants in the ABO gene were selected from post quality control data [15]; only common variants with minor allele frequencies (MAF) ≥0.05 were included in our analysis.

### Statistical analysis

Differences in patient and tumor characteristics across ABO blood types were examined with Student’s t tests and x^2^ tests. Hardy-Weinberg equilibrium (HWE) was evaluated by comparing observed and expected genotype frequencies. Associations between traits and genetic variants were assessed using exact tests. Associations with OS were evaluated using Cox proportional hazards regression in both unadjusted and multivariable adjusted models that included adjustment for age, race stage, grade, histologic subtype of disease, and year of diagnosis. For variants, additive and dominant genetic effect models were employed. Hazard ratios (HRs) and 95% confidence intervals (CIs) were used to quantify associations with OS, defined as the interval between the date of diagnosis and either death or last EMR entry. As more than half of women with ovarian cancer die within 5 years of diagnosis, and confidence intervals generally increase as sample size decreases over time in such analyses, we did not evaluate OS beyond 10 years. Interactions were evaluated with likelihood ratio tests of nested models. Survival functions were visualized using Kaplan-Meier plots; differences were evaluated with the Log-Rank test. All analyses were performed using SAS version 9.4 (SAS Institute, Cary, NC) and statistical significance was defined with a two-tailed threshold of 0.05.

## Results

ABO blood type (N = 694) and/or ABO genetic variants (N = 154) were available for a total of 713 Tumor Registry confirmed ovarian or fallopian tube cancer cases from the VUMC (**Table 1**). Cases were predominantly Caucasian (N = 637, 89.3%) and had a mean age at diagnosis of 58.7 years. As expected, the majority had advanced stage at diagnosis (Stage III & Stage IV: N = 422, 59.2%), high grade disease (G3 & G4: N = 380, 53.3%), and serous histology (N = 420, 58.9%). Blood type from EMR-linked laboratory assays included 312 type A (45.0%), 85 type B (12.2%), 39 type AB (5.6%), and 258 type O (37.2%). Blood type was not associated with any clinical covariate, with the exception of race; Caucasian cases were more likely to be blood type A or O, whereas cases of unknown and other races were more likely to be blood type B or AB (P-value = 0.002). When analysis was restricted to Caucasians, blood type was not associated with any patient or tumor characteristic evaluated (data not shown).

**Table 1:** Clinical characteristics and ABO blood type among Tumor Registry Confirmed ovarian cancer cases from VUMC EMR.

		All Phenotyped or Genotyped Cases (N = 713)		Among Cases with Blood Type Available from EMR-linked Laboratory Assays (N = 694)								
				Blood Type A (N = 312)		Blood Type B (N = 85)		Blood Type AB (N = 39)		Blood Type O (N = 258)		
Characteristic	N or mean	(% or std dev) ^a^	N or mean	(% or std dev) ^a^	N or mean	(% or std dev) ^a^	N or mean	(% or std dev) ^a^	N or mean	(% or std dev) ^a^	P-value ^b^
**Age at Diagnosis**, years	58.7	(13.6)	58.8	(13.8)	57.6	(14.3)	58.2	(13.3)	59.0	(13.5)	0.482
**Race**											
White	637	(89.3)	290	(93.0)	67	(78.8)	34	(87.2)	232	(89.9)	**0.002**
Other/Unknown	76	(10.7)	22	(7.1)	18	(21.2)	5	(12.8)	26	(10.1)
**Histologic Subtype**											
Serous	420	(58.9)	181	(58.0)	54	(63.5)	22	(56.4)	154	(59.7)	0.798
Non-Serous	178	(25.0)	81	(26.0)	22	(25.9)	11	(28.2)	59	(22.9)
Unknown	115	(16.1)	50	(16.0)	9	(10.6)	6	(15.4)	45	(17.4)
**Stage**											
I/II	176	(24.7)	79	(25.3)	24	(28.2)	9	(23.1)	61	(23.6)	0.616
III/IV	422	(59.2)	182	(58.3)	52	(61.2)	27	(69.2)	155	(60.1)
Unknown/Unstaged	115	(16.1)	51	(16.4)	9	(10.6)	3	(7.7)	42	(16.3)
**Grade**											
G1 & G2	138	(19.4)	61	(19.6)	25	(29.4)	8	(20.5)	41	(15.9)	0.186
G3 & G4	380	(53.3)	174	(55.8)	39	(45.9)	19	(48.7)	143	(55.4)
Unknown	195	(27.4)	77	(24.7)	21	(24.7)	12	(30.8)	74	(28.7)

^a^ Column percentages may not sum to 100% due to rounding error

^b^ Bold values denote significant associations

**Fig 1:**
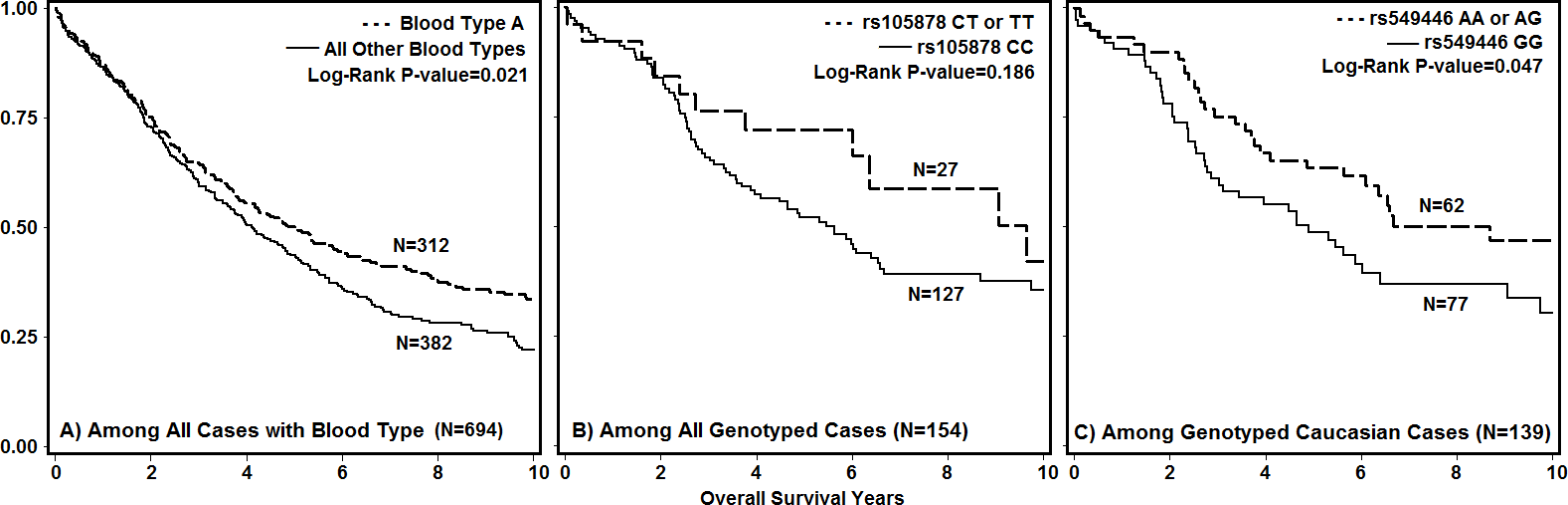
Kaplan-Meier survival functions for overall ovarian cancer survival. A) Blood type A vs all other types among all cases with blood type from linked EMR; 1B) Minor allele carriers of rs1053878 (A2) vs non-minor allele carriers among all genotyped cases; 1C) Minor allele carriers of rs549446 (r^2^>0.2 with rs505922) vs non-minor allele carriers among genotyped Caucasian cases; X-axis = years of Overall Survival; Y-axis = Percent of Cases Alive.

Cases with blood type A had significantly better OS in both unadjusted analyses, and after adjustment for clinical covariates and race (**Table 2**), either compared to blood type O (HR: 0.75, 95% CI: 0.60–0.93) or all non-A blood type cases (HR: 0.77, 95% CI: 0.63–0.94). Results from Kaplan-Meier analyses were in agreement (**Fig 1A**), showing significantly better OS for ovarian cancer cases with blood type A as compared to all other blood types by the Log-Rank test (P-value = 0.021). When analyses were stratified, results did not vary by histology, stage, or grade (all P-interaction>0.05), although associations among cases with serous histology (HR: 0.72, 95% CI: 0.55–0.94), late stage disease (HR: 0.76, 95% CI: 0.59–0.98), and high grade disease (HR: 0.71, 95% CI: 0.56–0.92) remained statistically significant, while the comparison of blood type A to O did not reach statistical significance among non-serous cases, early-stage cases, and cases with low grade disease (data not shown). The association between blood type and OS differed by race (P-interaction = 0.047), such that better survival for type A cases occurred only among Caucasian cases (HR: 0.72, 95% CI: 0.58–0.89) (**Table 2**). No association was found among cases of other ethnicities (HR: 1.60, 95% CI: 0.86–3.00), that included 38 Black, seven Asian, and two Native women; 24 cases of unknown race/ethnicity were excluded from race-stratified analyses.

**Table 2:** ABO blood type and overall ovarian cancer survival among Tumor Registry Confirmed cases from VUMC EMR.

		Cases			5 year Survival	Unadjusted Association ^a^			Adjusted Association ^a^^,^ ^b^		
		N	%	Deaths		HR ^a^	95% CI^a^	P-value	HR^a^^,^ ^b^	95% CI ^a^^,^ ^b^	P-value
**Among All Cases with ABO Phenotype**											
	O Phenotype	258	37.2%	172	44.6%	1.0 (reference)			1.0 (reference)		
	A Phenotype	312	45.0%	173	49.6%	**0.80**	**0.65–0.98**	**0.034**	**0.75**	**0.60–0.93**	**0.008**
	B Phenotype	85	12.2%	52	39.0%	0.98	0.72–1.33	0.872	0.94	0.69–1.28	0.687
	AB Phenotype	39	5.6%	26	38.5%	1.06	0.70–1.60	0.780	0.87	0.58–1.32	0.522
	All Others	382	55.0%	250	43.2%	1.0 (reference)			1.0 (reference)		
	A Phenotype	312	45.0%	173	49.6%	**0.80**	**0.66–0.97**	**0.021**	**0.77**	**0.63–0.94**	**0.009**
**Among Caucasian Cases with ABO Phenotype**											
	O Phenotype	232	37.2%	154	45.8%	1.0 (reference)			1.0 (reference)		
	A Phenotype	290	46.5%	154	50.8%	**0.77**	**0.62–0.96**	**0.021**	**0.70**	**0.56–0.88**	**0.002**
	B Phenotype	67	10.8%	42	36.0%	1.03	0.73–1.45	0.856	0.95	0.67–1.34	0.768
	AB Phenotype	34	5.5%	23	36.3%	1.14	0.74–1.77	0.560	0.88	0.56–1.37	0.563
	All Others	333	53.5%	219	43.5%	1.0 (reference)			1.0 (reference)		
	A Phenotype	290	46.5%	154	50.8%	**0.76**	**0.61–0.93**	**0.008**	**0.72**	**0.58–0.89**	**0.002**
**Among Non-Caucasian Cases with ABO Phenotype**											
	O Phenotype	26	36.6%	18	30.9%	1.0 (reference)			1.0 (reference)		
	A Phenotype	22	31.0%	19	31.8%	1.26	0.66–2.41	0.486	1.62	0.76–3.44	0.210
	B Phenotype	18	25.4%	10	46.4%	0.70	0.32–1.52	0.368	1.03	0.42–2.56	0.945
	AB Phenotype	5	7.0%	3	44.0%	0.61	0.18–2.08	0.431	0.99	0.25–3.96	0.985
	All Others	49	69.0%	31	40.3%	1.0 (reference)			1.0 (reference)		
	A Phenotype	22	31.0%	19	31.8%	1.51	0.85–2.68	0.158	1.60	0.86–3.00	0.140

^a^ Hazard Ratio (HR) and 95% Confidence Interval (CI) from proportional hazards regression; bold type denotes significant association

^b^ Adjusted for age, stage, race, histologic subtype, grade, and year of diagnosis

To delve further into the relationship between blood type and ovarian cancer survival, we also examined linked genetic data. Ten common (MAF>0.05) variants in the ABO gene were included on the Illumina Exome BeadChip, passed quality control, and were available for analysis for 154 VUMC cases (**Table 3**). Half of the variants (rs549446, rs8176720, rs8176740, rs8176745, and rs8176746) were found to deviate from HWE. All ten variants were significantly associated with blood type (all P-values≤0.027). No variants were significantly associated with race, and HWE and ABO phenotype associations were unchanged when analyses were restricted to Caucasian cases (data not shown). Based on information compiled from dbSNP [2], OMIM [3], and published literature [4–6], common variants related to ABO phenotype included rs1053878 (A2), rs8176746 (B), and rs512770 (O2), as well as four variants (rs549446, rs8176740, rs8176742, and rs8176745) with moderate (r^2^>0.2) linkage disequilibrium (LD) with rs505922, which has been reported to be in perfect LD with rs8176719 (O), plus three additional synonymous variants (rs8176720, rs8176741, and rs8176749) for which no phenotypic relevance was identified.

**Table 3:** Common ABO variants evaluated and phenotype and related information.

					All Genotyped Cases (N = 154)				ABO Phenotype
Variant	Chr 9 Location	Variant Location, Type	Amino Acid	Alleles ^a^	MAF ^b^	HWE ^c^	ABO ^d^	Race ^d^	Information ^e^
rs549446	133259834	Exon 4, Missense	63 (Arg > His)	G/A	0.240	**0.031**	**0.002**	0.394	r^2^ with rs505922 = 0.218
rs512770	133258116	Exon 5, Missense	74 (Pro > Ser)	C/T	0.195	0.144	**0.009**	0.074	minor allele = O2
rs8176720	133257486	Exon 6, Synonymous	99 (Thr)	A/G	0.325	**0.022**	**<0.001**	0.231	
rs1053878	133256264	Exon 7, Missense	156 (Pro > Leu)	C/T	0.094	0.549	**<0.001**	0.294	minor allele = A2
rs8176740	133256085	Exon 7, Missense	216 (Phe > Ile)	T/A	0.234	**0.004**	**<0.001**	0.664	r^2^ with rs505922 = 0.218
rs8176741	133256074	Exon 7, Synonymous	219 (His)	C/T	0.075	0.317	**<0.001**	0.243	
rs8176742	133256050	Exon 7, Synonymous	227 (Pro)	G/A	0.232	**0.005**	**0.001**	0.818	r^2^ with rs505922 = 0.218
rs8176745	133255960	Exon 7, Synonymous	257 (Pro)	C/T	0.234	**0.004**	**<0.001**	0.664	r^2^ with rs505922 = 0.218
rs8176746	133255935	Exon 7, Missense	266 (Leu > Met)	C/A	0.075	0.317	**<0.001**	0.243	minor allele = B
rs8176749	133255801	Exon 7, Synonymous	310 (Leu)	G/A	0.078	0.942	**<0.001**	0.309	

^a^ Major/minor allele on the coding (reverse) strand

^b^ Minor allele Frequency (MAF)

^c^ Hardy-Weinberg equilibrium p-value; bold value denotes significant disequilibrium

^d^ p-value from exact test with ABO phenotype or race (dichotomized); bold value denotes significant association

^e^ O variant rs8176719 not included in HapMap or 1000G, but reported to be in perfect linkage disequilibrium with rs505922

Associations between ABO variants and OS were first evaluated with additive models, but as the number of cases homozygous for any variant was small (range: 0–10, mean = 2.6), we also employed dominant models (**Table 4**). The A2 variant (rs1053878) was associated with significantly better OS (HR: 0.50, 95% CI 0.25–0.99), but only in multivariable adjusted models among all cases, including 139 Caucasian, nine unknown, and six Black cases. The four variants in moderate LD with rs505922—and therefore also rs8176719 (O)—were associated with better survival, but only in unadjusted analyses among Caucasian cases (HR: 0.62, 95% CI: 0.39–0.99). Kaplan-Meier analyses were in general agreement, with non-significantly better OS (Log-Rank P-value = 0.186) for minor allele carriers of rs1053878 (**Fig 1B**), and significantly better OS (Log-Rank P-value = 0.047) for Caucasian minor alleles carriers of rs549446 (**Fig 1C**). Due to limited genetic data, diplotype analysis and analysis among non-Caucasian cases was precluded.

**Table 4:** Common ABO variants and overall ovarian cancer survival among Tumor Registry Confirmed cases from VUMC EMR.

				Additive Genetic Models (per minor allele)						Dominant Genetic Models (any minor allele)					
	N Cases / N Deaths ^a^			Unadjusted Association ^b^			Adjusted Association ^b^^,^ ^c^			Unadjusted Association ^b^			Adjusted Association ^b^^,^ ^c^		
	0	1	2	HR^b^	95% CI ^b^	P-value	HR ^b^^,^ ^c^	95% CI ^b^^,^ ^c^	P-value	HR^b^	95% CI ^b^	P-value	HR^b^^,^^c^	95% CI ^b^^,^ ^c^	P-value
**Among All Cases (N = 154)**															
rs549446	84 / 49	66 / 32	4 / 2	0.70	0.46–1.04	0.078	0.82	0.53–1.27	0.374	0.67	0.43–1.04	0.071	0.79	0.49–1.27	0.950
rs512770	97 / 53	54 / 27	3 / 3	0.89	0.59–1.36	0.597	1.15	0.75–1.76	0.528	0.81	0.52–1.27	0.353	1.06	0.66–1.70	0.802
rs8176720	64 / 37	80 / 39	10 / 7	0.85	0.59–1.24	0.404	1.05	0.70–1.58	0.800	0.73	0.48–1.13	0.160	0.88	0.55–1.40	0.580
rs1053878	127 / 72	25 / 10	2 / 1	0.69	0.39–1.22	0.200	0.55	0.29–1.04	0.066	0.65	0.35–1.23	0.190	**0.50**	**0.25–0.99**	**0.045**
rs8176740	84 / 49	68 / 33	2 / 1	0.68	0.45–1.04	0.073	0.82	0.51–1.30	0.390	0.67	0.43–1.04	0.071	0.79	0.49–1.27	0.330
rs8176741	131 / 69	23 / 14	0 / 0	1.23	0.69–2.19	0.477	1.14	0.63–2.04	0.667	1.23	0.69–2.19	0.506	1.14	0.63–2.04	0.667
rs8176742	84 / 49	67 / 32	2 / 1	0.67	0.44–1.02	0.062	0.80	0.50–1.29	0.363	0.65	0.42–1.02	0.059	0.78	0.48–1.26	0.304
rs8176745	84 / 49	68 / 33	2 / 1	0.68	0.45–1.04	0.073	0.82	0.51–1.30	0.390	0.67	0.43–1.04	0.071	0.79	0.49–1.27	0.330
rs8176746	131 / 69	23 / 14	0 / 0	1.23	0.69–2.19	0.477	1.14	0.63–2.04	0.667	1.23	0.69–2.19	0.477	1.14	0.63–2.04	0.667
rs8176749	131 / 69	22 / 14	1 / 0	1.10	0.65–1.86	0.725	1.12	0.63–2.00	0.697	1.23	0.69–2.19	0.506	1.14	0.63–2.04	0.667
**Among Causasian Cases (N = 139)**															
rs549446	77 / 44	59 / 28	3 / 1	**0.63**	**0.41–0.98**	**0.040**	0.74	0.46–1.20	0.226	**0.62**	**0.39–0.99**	**0.049**	0.73	0.44–1.21	0.221
rs512770	91 / 48	46 / 23	2 / 2	0.87	0.55–1.37	0.555	1.09	0.68–1.75	0.714	0.81	0.50–1.32	0.395	1.02	0.61–1.68	0.954
rs8176720	60 / 34	71 / 34	8 / 5	0.79	0.53–1.18	0.248	0.92	0.60–1.42	0.709	0.70	0.44–1.11	0.128	0.79	0.48–1.28	0.329
rs1053878	115 / 64	23 / 9	1 / 0	0.65	0.33–1.28	0.214	0.62	0.29–1.31	0.208	0.67	0.33–1.34	0.251	0.62	0.29–1.31	0.212
rs8176740	77 / 44	60 / 28	2 / 1	0.64	0.41–1.01	0.053	0.76	0.46–1.24	0.273	**0.62**	**0.39–0.99**	**0.049**	0.73	0.44–1.21	0.221
rs8176741	120 / 63	19 / 10	0 / 0	1.02	0.52–1.99	0.957	0.87	0.44–1.72	0.684	1.02	0.52–1.99	0.957	0.87	0.44–1.72	0.684
rs8176742	77 / 44	60 / 28	2 / 1	0.64	0.41–1.01	0.053	0.76	0.46–1.24	0.273	**0.62**	**0.39–0.99**	**0.049**	0.73	0.44–1.21	0.221
rs8176745	77 / 44	60 / 28	2 / 1	0.64	0.41–1.01	0.053	0.76	0.46–1.24	0.273	**0.62**	**0.39–0.99**	**0.049**	0.73	0.44–1.21	0.221
rs8176746	120 / 63	19 / 10	0 / 0	1.02	0.52–1.99	0.957	0.87	0.44–1.72	0.684	1.02	0.52–1.99	0.957	0.87	0.44–1.72	0.684
rs8176749	120 / 63	18 / 10	1 / 0	0.93	0.50–1.71	0.810	0.86	0.44–1.69	0.659	1.02	0.52–1.99	0.957	0.87	0.44–1.72	0.684

^a^ N cases / N deaths for cases with no minor alleles (0), heterozygotes (1), and minor allele homozygotes (2)

^b^ Hazard Ratio (HR) and 95% Confidence Interval (CI) from proportional hazards regression; bold text denotes significant association

^c^ Adjusted for age, stage, grade, histologic subtype, and year of diagnosis

## Discussion

In this large single-institution retrospective study of Tumor Registry, EMR, laboratory, and genetic data, we found that ovarian cancer cases with blood type A had approximately 20% longer OS than other cases. Our phenotype sample size (N = 694) is the largest to date of studies on blood type and ovarian cancer survival, and we found statistically significant associations among all cases and among Caucasian cases. Despite a limited number of genotyped cases (N = 154), we also found suggestive associations with ABO variants. First, cases with minor alleles of rs1053878, which distinguishes the A1 and A2 alleles, had a 50% lower risk of death. This agrees with our phenotype results showing better survival for cases with blood type A, and implies that the association may be driven by the A2 allele. Second, Caucasian cases with minor alleles of any of four variants in perfect LD (r^2^ = 1) had a 38% lower risk of death, but significance was attenuated after multivariable adjustment. These four variants (rs549446, rs8176740, rs8176742, and rs8176745) share moderate LD with rs505922, which is in perfect LD with the protein truncating O variant (rs8176719), although the O phenotype was not related to overall ovarian cancer survival in our analysis.

Early studies suggested an association between blood type and ovarian cancer risk, with higher frequencies of type A among women with ovarian cancer than type O [16,17]. More recently, a meta-analysis of eight OCAC studies with 5,233 cases and 6,837 controls indicated that women with genetic variants corresponding to blood type A had 9% greater ovarian cancer risk than women with variants corresponding to type O [8]. Notably, increased risk was only evident for A1 and not A2 genotype cases [8]. Similar to prior studies, we also found a higher prevalence of type A than type O cases among Caucasians our analysis. Further, half of the ABO variants evaluated were found to deviate from HWE. While not definitive, these two findings are generally supportive of a relationship between blood type and ovarian cancer risk. With regards to ovarian cancer survival, prior studies on blood type are limited. One small Italian study reported better 5-year survival for type O cases, but included only 92 ovarian cases and did not conduct regression analysis [9]. Among 256 Chinese women, blood type A was associated with significantly worse overall survival (HR: 2.24, 95% CI: 1.36–3.67) in analyses that included adjustment for age, grade, and stage [10]. As these studies contradict our current findings, we sought possible explanations. First, we excluded stage IV cases and amended our adjustment to be comparable to the Chinese study, but still found better survival for type A compared to type O cases (HR: 0.69, 95% CI: 0.54–0.88). Second, we compared the prevalence of blood types in both study and source populations (**S1 Table**). Our prevalence of type A was higher than Caucasians across the US, while the prevalence of type A among the 256 Chinese cases was lower than found across China. Thus, selection of ovarian cancer cases might contribute to non-representative study populations, and differences in ABO blood type by race/ethnicity could also add to differences in findings.

Results for ABO blood type and survival from other solid malignancies have also been mixed. For pancreatic cancer, blood type O had longer median survival in one study of 316 resected Chinese cases [18], and 22% better survival among 576 resected German cases [19]. On the contrary, blood type was not associated with pancreatic cancer survival among 488 Chinese cases [20], and ABO variants were not associated with survival among 1,028 cases from the PANcreatic Disease ReseArch (PANDoRA) consortium, although analysis was not adjusted for clinical covariates [6]. Breast cancer survival results also vary. Some studies have reported significant associations, such as a lower risk of death for blood type O among 315 cases [21], longer survival for blood types A and O among 335 non-metastatic cases [22], and higher rates of recurrence and death for blood types B and AB among 1,001 invasive cases [23]. However, no association between blood type and breast cancer mortality was seen among 426 cases [24], 468 triple-negative cases [25], and 2,036 cases from the Nurses’ Health Study [26]. Since the majority of existing studies have evaluated only phenotype, rather than ABO genotype, we speculate that some of the inconsistencies across prior studies may arise from differences in allelic proportions of A1/A2 and O1/O2 across diverse populations.

Multiple potential mechanisms to support a role for ABO blood type in cancer development and progression have been proposed. The ABO gene encodes a glycosyltransferase, and ABO antigens are expressed not only on RBCs but also on endothelial and epithelial cells. Dysregulation of ABO enzymatic activity could influence cellular adhesion, cell membrane signaling, and host immune response [13]. Aberrant ABO antigens have been found on tumor tissues compared to normal cells, such as loss of the A or B epitope and accumulation of the H antigen, or incompatible expression of A antigens by tumors in subjects with blood type O [11]. Altered ABO expression may contribute to a malignant phenotype via improved cell motility or enhanced evasion of apoptosis [12]. ABO variants have also been linked to several mediators of inflammation and immune cell recruitment cascades by GWAS, including endothelial leukocyte adhesion molecule, also known as E-selectin, platelet alpha-granule membrane protein, also known as P-selectin [27–30], and soluble intercellular adhesion molecule (ICAM)-1. E-selectin mediates the accumulation of leukocytes during inflammation, and P-selectin enables leukocytes to interact with platelets or activated endothelial cells. ICAM-1 is rapidly upregulated by inflammatory cytokines and can block lymphocyte adhesion to endothelial cells [31]. Both P-selection and ICAM-1 are associated with the A1 allele of the ABO blood group [30], which was shown to be associated with increased ovarian cancer risk by OCAC [8].

In addition to antigen and immune dysregulation, ABO blood type may influence ovarian cancer survival by mechanisms related to coagulation. The ABO gene product is responsible for post-translational glycosylation of Von Willebrand factor (vWF), a procoagulant molecule involved in platelet adhesion [32]. Venous thromboembolism (VTE) has been associated with poor ovarian cancer survival [33,34], and patients with blood type O have lower rates of VTE, possibly due to lower levels of vWF [35]. Recent evidence also suggests that platelets may play a larger role in VTE than previously thought [36]. Paraneoplastic thrombocytosis, or elevated platelet levels driven by cancer progression, is a long-recognized phenomenon [37]. Pre-diagnosis thrombocytosis has been associated with reduced ovarian cancer survival [38]; postulated mechanisms include paracrine mediators and cytokine communication between platelets, liver, bone marrow, and ovarian cancer cells [39]. Thus, thrombocytosis and VTE are additional ways that blood type may influence ovarian cancer survival. Intriguingly, while GWAS have shown that individuals with blood type O have lower VTE risk and lower vWF levels than non-O blood types, the A2 blood type was found to be independently associated with lower VTE risk, and to have the lowest vWF levels of all blood types [40,41]. Thus, better survival among ovarian cancer cases with blood type A is biologically plausible, and could be due to the A2 allele.

Strengths of this study include a large study population of 713 Tumor Registry confirmed cancer cases from a single tertiary-care medical center. In contrast, two prior reports of blood type and ovarian cancer survival included only 92 and 256 cases [9,10]. Additional strengths include evaluation of both blood type and genetic variation in the ABO gene, and employment of appropriate statistical methods, such as multivariable adjustment for known prognostic factors in regression models. Limitations include that our cases were diagnosed over 33 years, during which time the definitions of optimal debulking [42] and histological classification have changed [43]. We did not adjust for surgical debulking, as this information was not readily available, but did include adjustment for serous or non-serous histology and calendar year of diagnosis. Our largest limitation is that genotype data was available for only 154 cases and included only ABO variants that were included on the Exome Beadchip. This platform was designed to capture potentially functional variants within coding regions, rather than providing comprehensive coverage across the genome. We did not have data for the single nucleotide deletion that results in protein truncation and the O phenotype (rs8176719). As this variant is not included in HapMap or 1000G, we could not directly assess LD; however, rs505922 has been reported to be a perfect proxy for rs8176719 (r^2^ = 1) [4–6], and in Caucasian populations, this variant has moderate LD (r^2^ = 0.218) with four variants that we did evaluate. Given the limited genetic data available, we were also unable to evaluate diplotypes. Thus, further study of ABO genotypes in relation to ovarian cancer outcomes should be undertaken, for example, by OCAC. Finally, race/ethnicity was administratively-assigned in our EMR. This has been shown to be as good as self-report [14], but as our population predominantly included Caucasians, generalizability of our results to other racial/ethnic groups may be limited.

In conclusion, ovarian cancer cases with blood type A had more favorable survival. This was evident whether type A was compared to type O or to all other blood types. The associations seemed to be driven by the A2 allele, although variants related to the O phenotype also had suggestive associations. Additional research is warranted to either replicate or refute our findings, and ultimately, to determine if ABO variants and blood type are causally related to ovarian cancer development, progression, and survival.

## Supporting information

S1 Table ABO blood type and prevalence information. (DOCX)
