# Correction: Blood type, ABO genetic variants, and ovarian cancer survival

**DOI:** 10.1371/journal.pone.0178965

**Published:** 2017-05-30

**Authors:** The PLOS ONE Staff

## Notice of Republication

This article was republished on May 8, 2017, to correct an error where an incorrect Fig 1 was published. The publisher apologizes for the error. Please download this article again to view the correct version.
