# Supplementary material for: Blood type, ABO genetic variants, and ovarian cancer survival
Source: PLoS One. 2017 Apr 27;12(4):e0175119. doi: 10.1371/journal.pone.0175119 (PMC5407760; doi:10.1371/journal.pone.0175119)
Supplement: S1 Table — (DOCX) [file pone.0175119.s001.docx]

**S1 Table. ABO Blood Type and Prevalence Information**
